# Datasets on agromorphological characters and distribution of cassava (Manihot esculenta L.) accessions cultivated in South-West and North-Central regions of Nigeria

**DOI:** 10.1016/j.dib.2024.110899

**Published:** 2024-09-03

**Authors:** Isaac O. Abegunde, Oghenevwairhe P. Efekemo, Olabode Onile-ere, Folashade Otitolaye, Emmanuel O. Idehen, Angela O. Eni

**Affiliations:** a Central and West African Virus Epidemiology Program, Covenant University Hub, Km. 10 Idiroko Road, Canaan Land, Ota, Ogun State, Nigeria; b Centre of Excellence in Agricultural Development and Sustainable Environment, Federal University of Agriculture, Abeokuta, P.M.B 2240, Alabata road, Ogun state, Nigeria; c Department of Biological Sciences, Covenant University, Km. 10, Idiroko Road Ota, Ogun State, Nigeria; d Department of Plant Breeding and Seed Technology, Federal University of Agriculture, Abeokuta, P.M.B 2240, Alabata road, Ogun state, Nigeria; e Regional Center of Excellence for Transboundary Plant Pathogens (Central and West African Virus Epidemiology, WAVE), Université Felix Houphouët-Boigny (UFHB), 01 BPV 34 Abidjan 01, Côte d'Ivoire

**Keywords:** Cassava traits, Morphological diversity, Food security, Epidemiological survey, Plant breeding, Crop improvement

## Abstract

The data presented here are agronomic indicators of cassava accessions collected during epidemiological surveys of cassava farms across South-West and North-Central Nigeria in 2021-2022. Cassava accessions were obtained from each farm surveyed and initially established in a randomized plot design at the WAVE Covenant University demonstration plot. Agronomic indicators were collected at 3-month intervals following the methods of (Fukuda et al., 2010) [1].

Specifications Table

**Table:** 

Subject	Agriculture and Plant Production
Specific subject area	Diversity and distribution of agronomic characters of cassava accessions collected from South-West and North-Central regions of Nigeria.
Type of data	Figures and Tables Raw
Data collection	The data were collected during cassava field surveys conducted across South-West and North-Central Nigeria in 2021-2022. Unique cassava cultivars were sampled from farms along a predetermined survey route at 10km intervals following previously described method by (Sseruwagi et al., 2004) [2]. Cultivars were collected and initially established on a demonstration plot and then scored using standard agronomic indicators.
Data source location	Data were collected from a demonstration plot at Covenant University (N 6° 40′ 18.5628", E 3° 9′ 29.25")
Data accessibility	Repository name: Mendeley data Data identification number: 10.17632/gh2nfyyknj.1 Direct URL to data: https://data.mendeley.com/datasets/gh2nfyyknj/1

## 1 Value of the Data

The agronomic data provided here are descriptions of agromorphological characters observed on cassava (Manihot esculenta L.) accessions collected for diversity studies from South-West and North-Central regions of Nigeria.

• The dataset could serve as a vital resource for evaluating progress made on interventions centered around the distribution and adoption of improved cassava varieties.
• The data serves as a preliminary resource for future in-depth diversity studies, where future research can utilize these available data to explore the characteristics and genetic potential of their collected accessions in greater details.
• These measured characteristics provide a baseline understanding of the various accessions collected in the survey regions, as the data provides insights into distinctive qualities each accession collected has, the distribution patterns of the different accessions across the surveyed regions and help understand farmers preferences by virtue of the qualities.
• Furthermore, it holds significance for educational purposes and serves as a valuable knowledge bank for breeding studies. The datasets also serve as a resource base for validation of other studies in selection, ultimately supporting agricultural sustainability and food security efforts.

## 2 Background

Cassava is an important food security crop in Nigeria given its resilience to changing climatic conditions, high carbohydrate content and the ease at which it is grown (Anyaegbu et al., 2023) [3]. Despite its importance to food security in Nigeria, farmers on the continent continue to suffer significantly lower yields compared to other cassava growing regions. The poor yields recorded on the continent are the result of poor farming practices, poor adoption of improved planting materials, pests and diseases. Current efforts to improve the productivity of cassava are primarily hinged on the adoption of improved planting materials. While this approach has been shown to be highly effective for several crops and across several regions of the world, its efficacy is often undermined by the lack of data on adoption, which is an essential requirement for ensuring effective interventions. While there have been a few studies assessing the diversity of cassava accessions cultivated in the country, only one study (Wossen et al., 2017) [4] has been conducted on the national or subnational scale. This dataset presented here provides an update to existing data on the diversity of cassava accessions across two important cassava growing regions of Nigeria. The dataset could serve as a vital first resource for evaluating progress made on interventions centred around the distribution and adoption of improved cassava varieties (Table 4, Figs. 25 and 26).

## 3 Data Description

The dataset is provided as an excel file with 3 sheets as outlined in Table 1 below. In summary, the dataset contains agronomic characteristics for a total of 470 cassava accessions collected across 290 fields as shown in Fig. 1. The distribution of the observed traits is presented in Fig. 2, Fig. 3, Fig. 4, Fig. 5, Fig. 6, Fig. 7, Fig. 8, Fig. 9, Fig. 10, Fig. 11, Fig. 12, Fig. 13, Fig. 14, Fig. 15, Fig. 16, Fig. 17, Fig. 18, Fig. 19, Fig. 20, Fig. 21, Fig. 22, Fig. 23, Fig. 24.

**Table 1:** Description of dataset.

Sheet Name	Description
Pre-Harvest	This sheet contains long form data of all traits observed prior to harvest.
Harvest	This sheet contains long form data for traits observed after accessions were harvested (12 MAP). The data presented are only for accessions that survived the entire study period.
MetaData	This sheet contains detailed descriptions for all variables in the two previous sheets

**Fig. 1:**
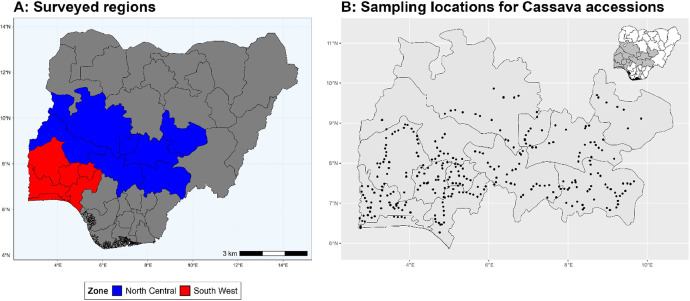
Locations from which cassava accessions were collected.

**Fig. 2:**
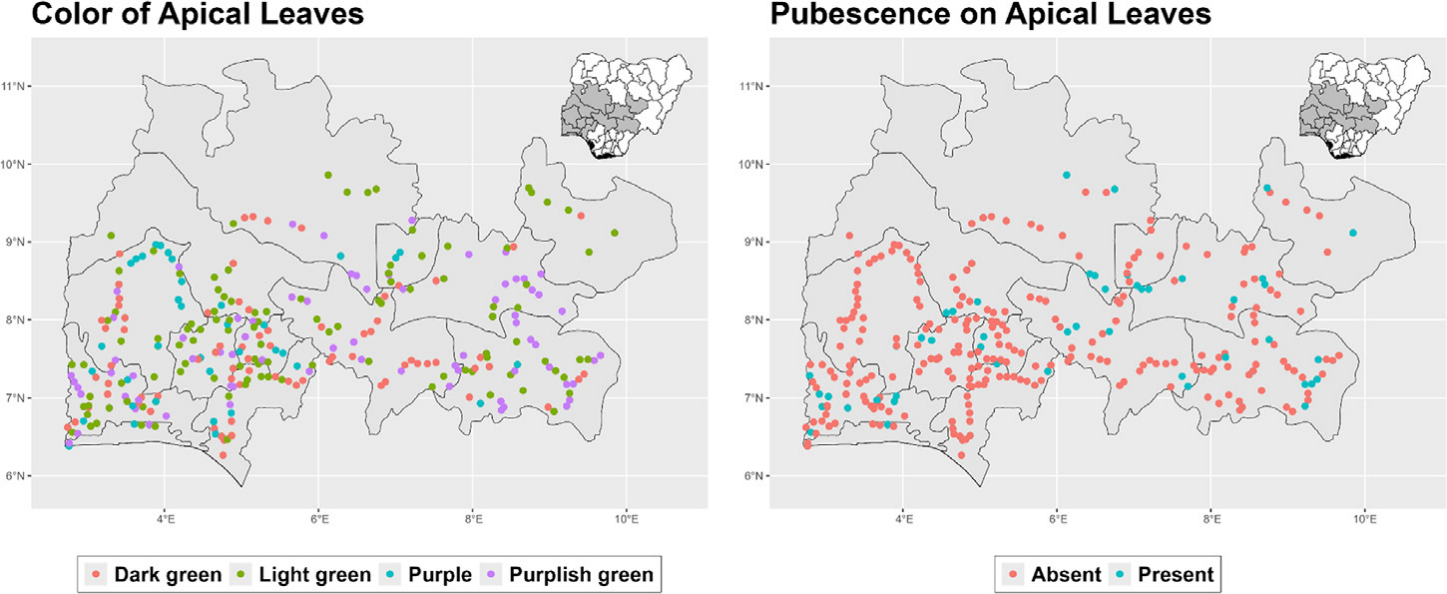
Variations in agromorphological traits scored at 3 months.

**Fig. 3:**
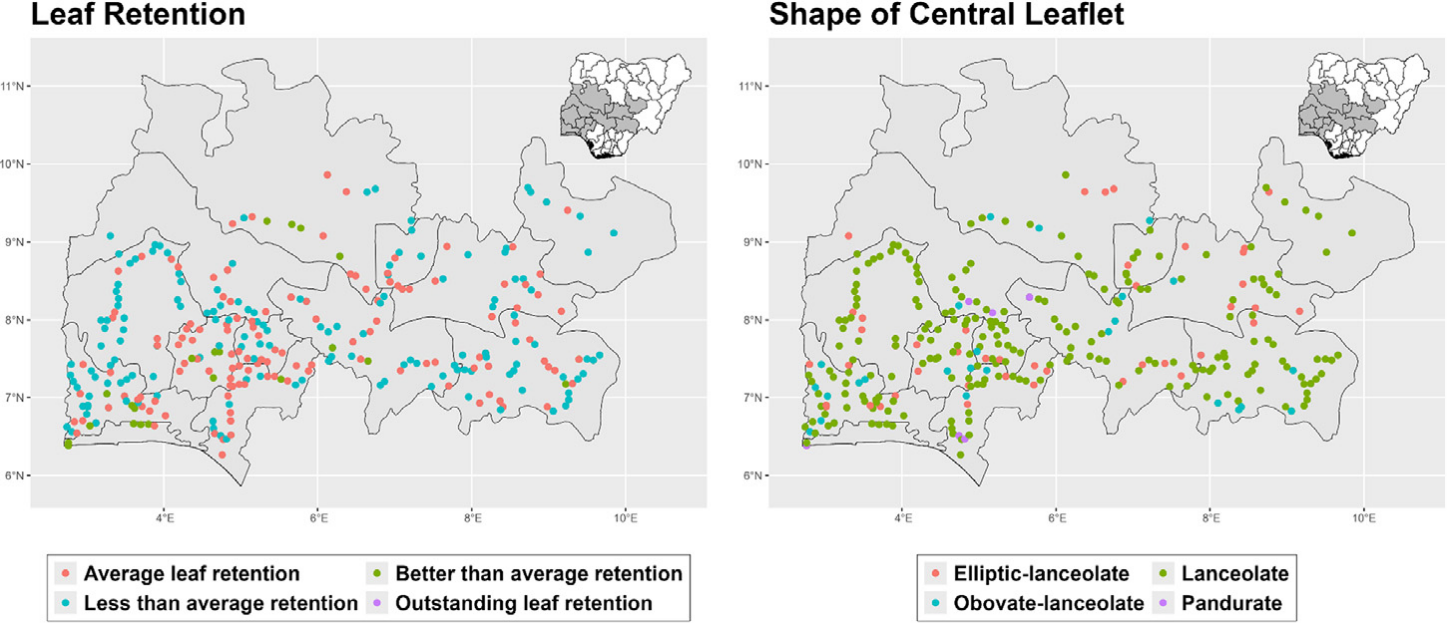
Variations in agromorphological traits scored at 6 months.

**Fig. 4:**
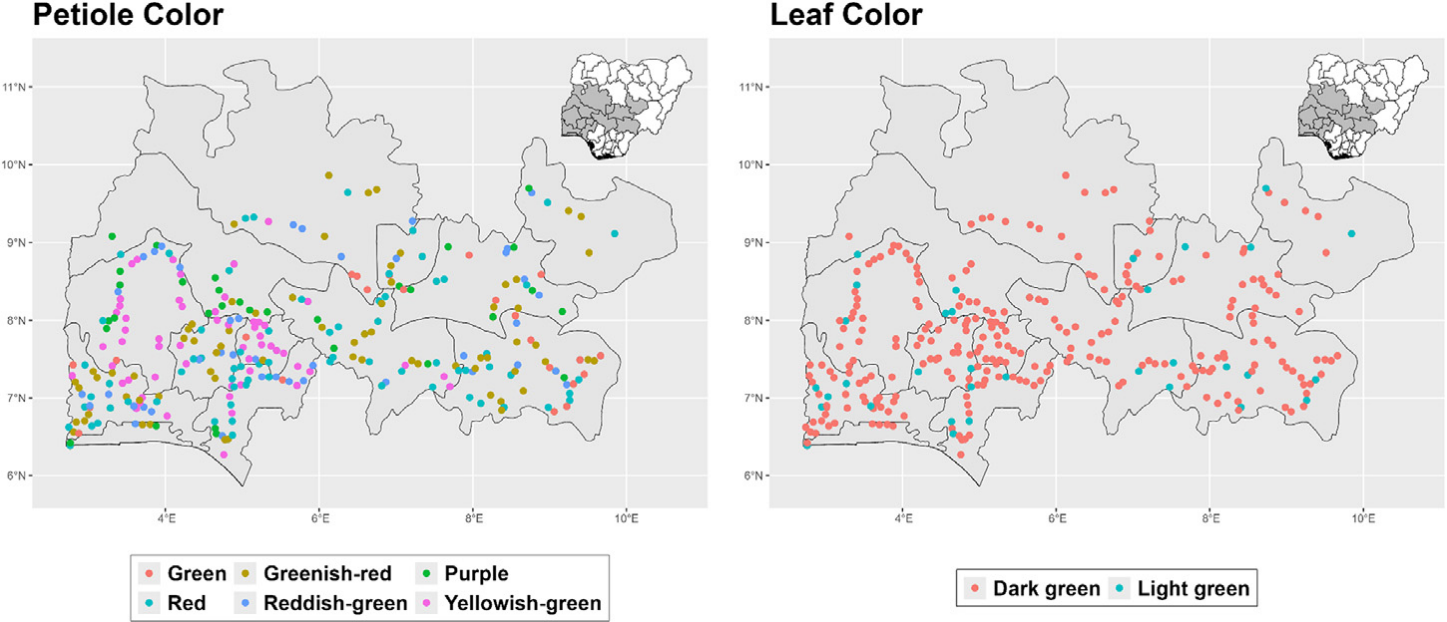
Variations in agromorphological traits scored at 6 months.

**Fig. 5:**
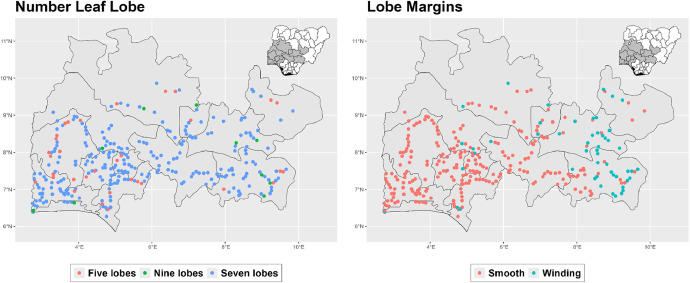
Variations in agromorphological traits scored at 6 months.

**Fig. 6:**
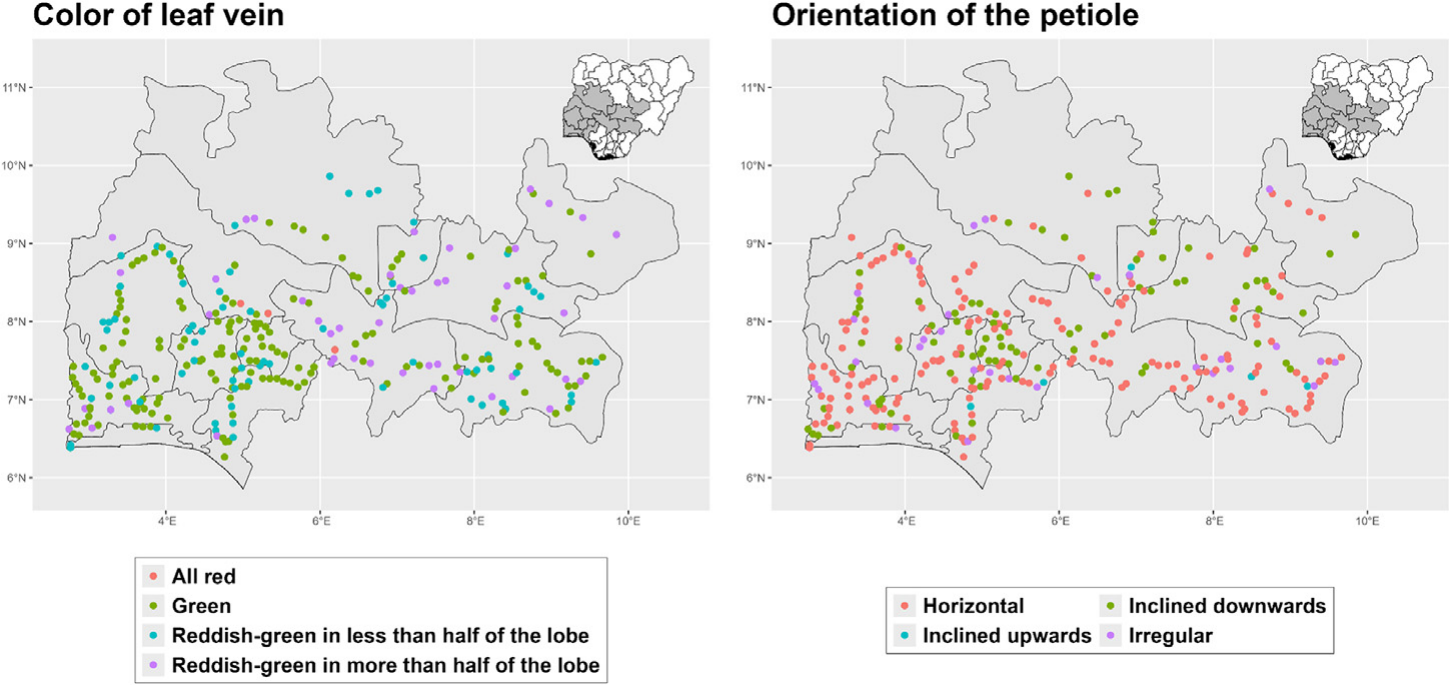
Variations in agromorphological traits scored at 6 months.

**Fig. 7:**
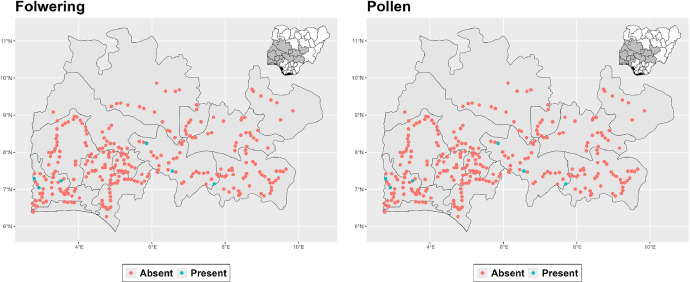
Variations in agromorphological traits scored at 6 months.

**Fig. 8:**
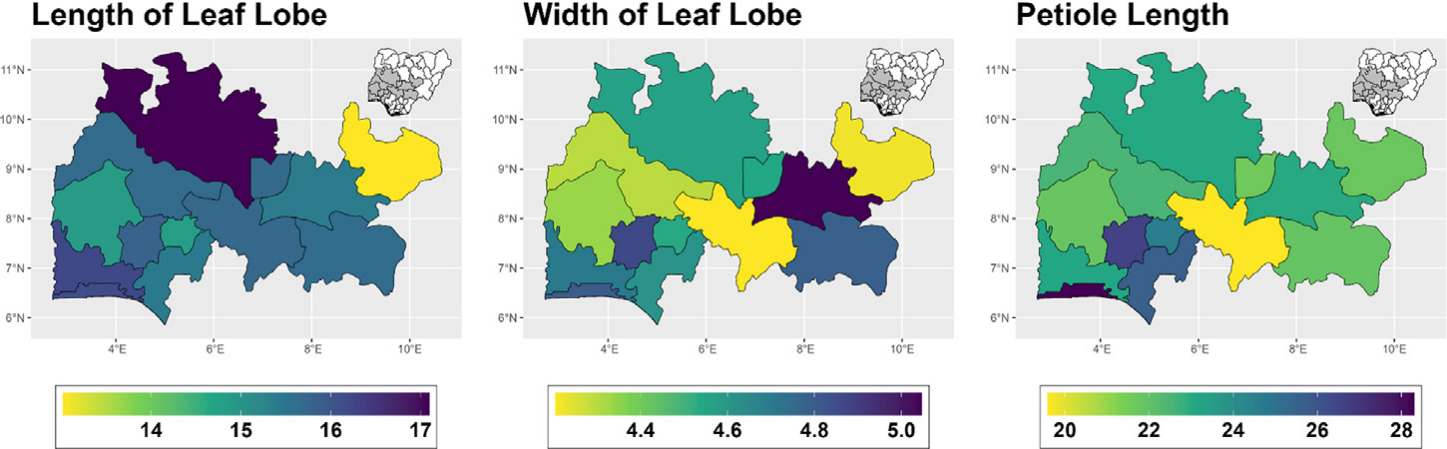
Variations in agromorphological traits scored at 6 months.

**Fig. 9:**
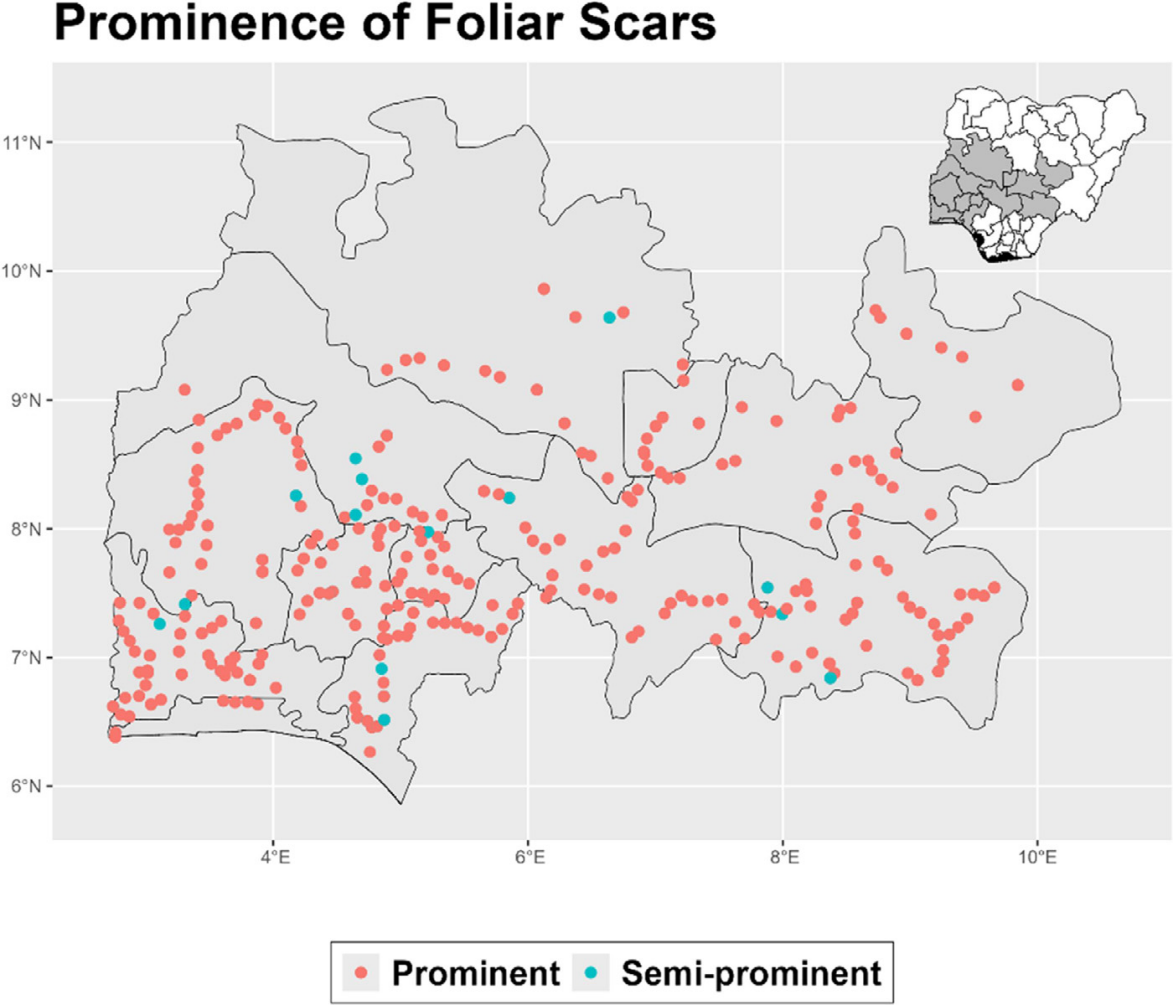
Variations in agromorphological traits scored at 9 months.

**Fig. 10:**
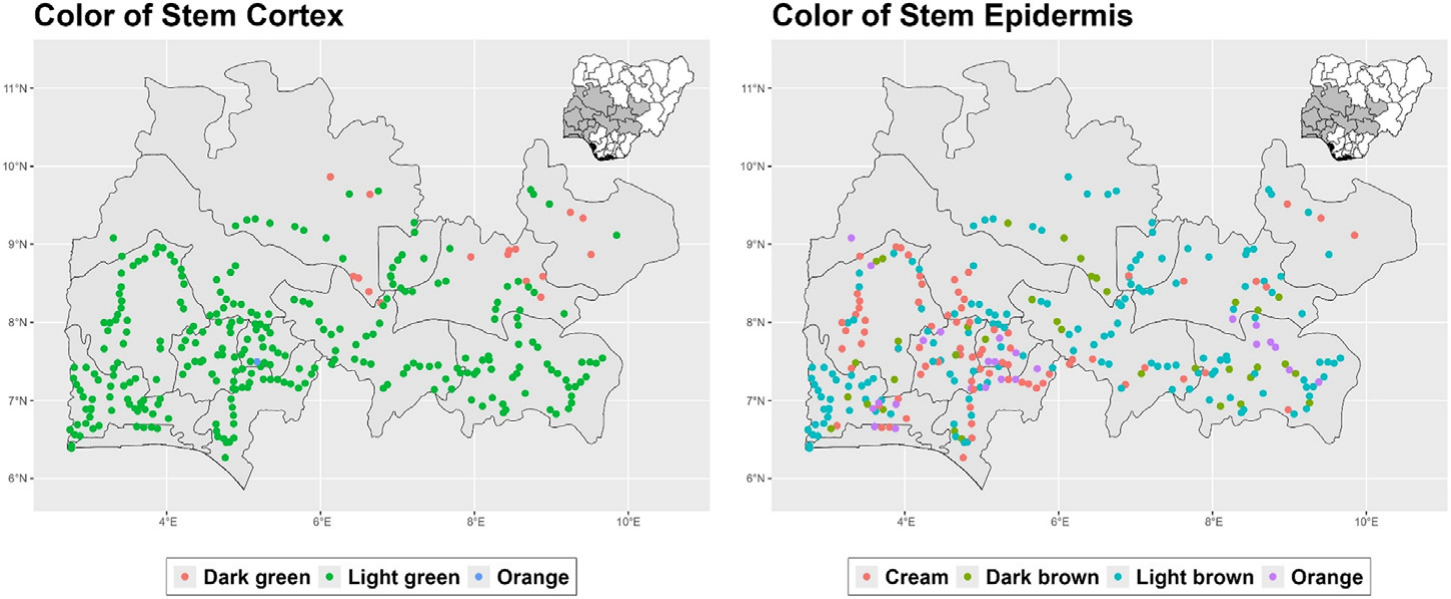
Variations in agromorphological traits scored at 9 months.

**Fig. 11:**
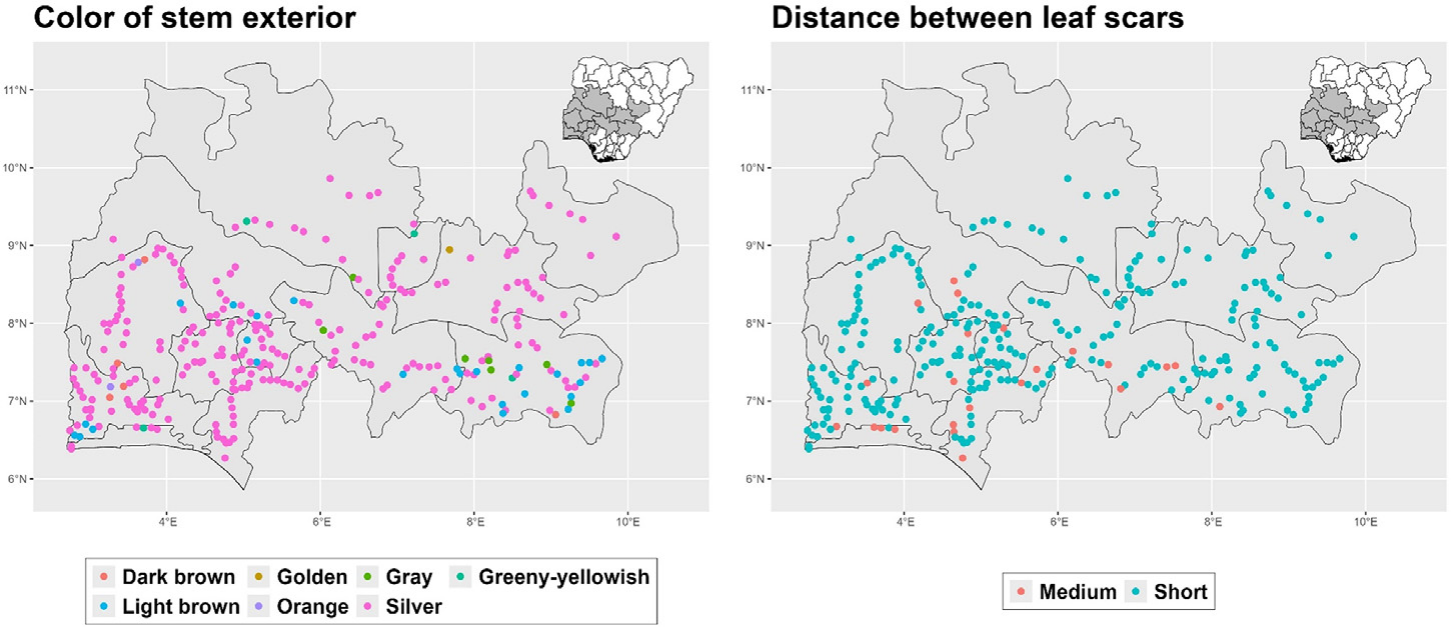
Variations in agromorphological traits scored at 9 months.

**Fig. 12:**
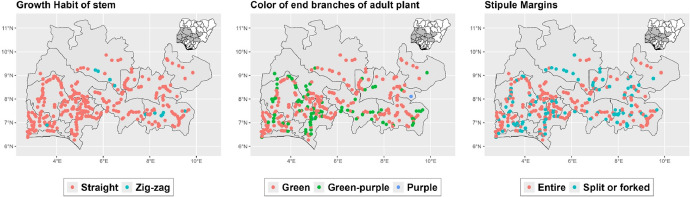
Variations in agromorphological traits scored at 9 months.

**Fig. 13:**
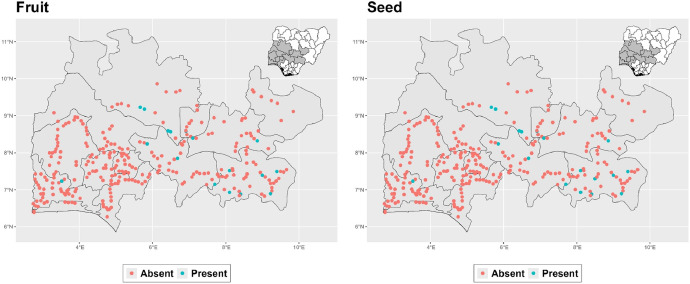
Variations in agromorphological traits scored at 12 months.

**Fig. 14:**
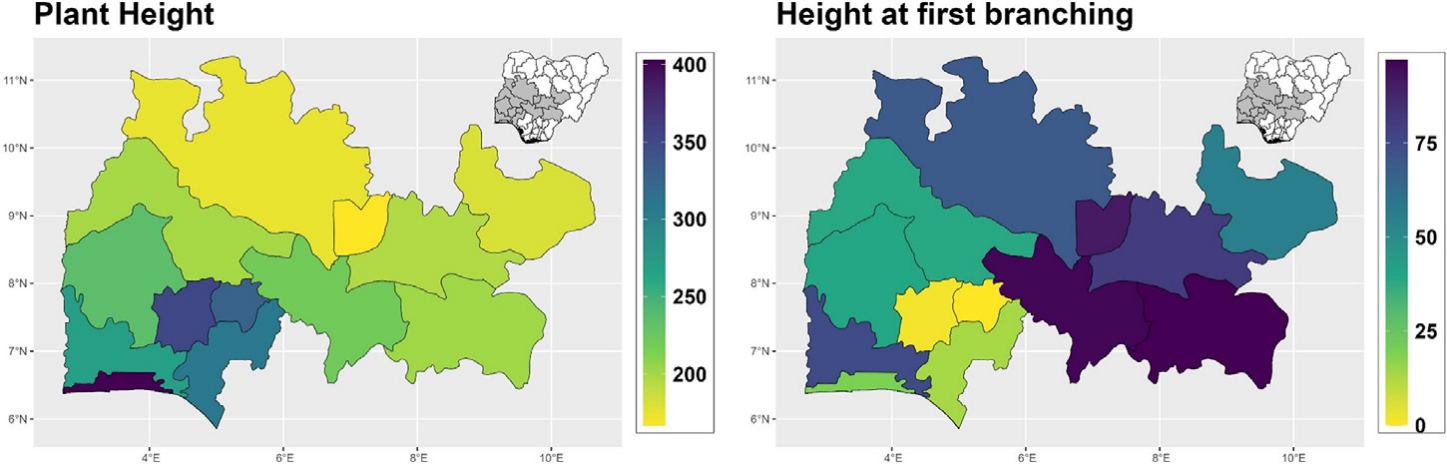
Variations in agromorphological traits scored at 12 months.

**Fig. 15:**
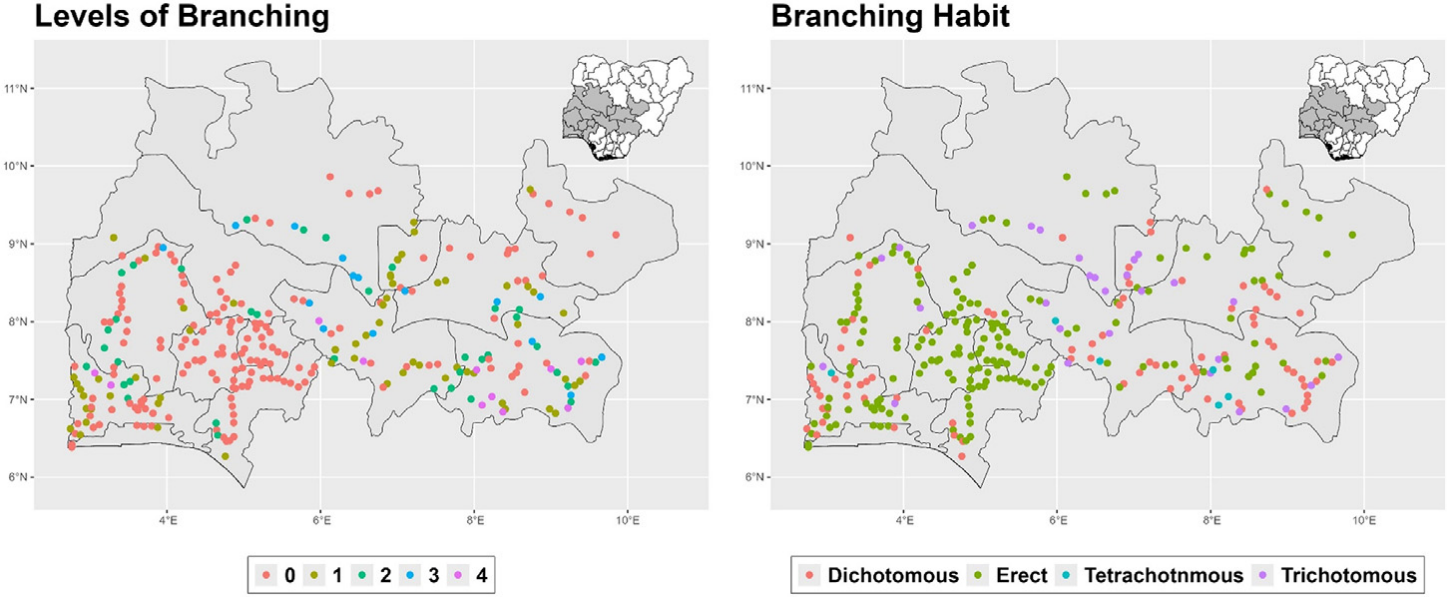
Variations in agromorphological traits scored at 12 months.

**Fig. 16:**
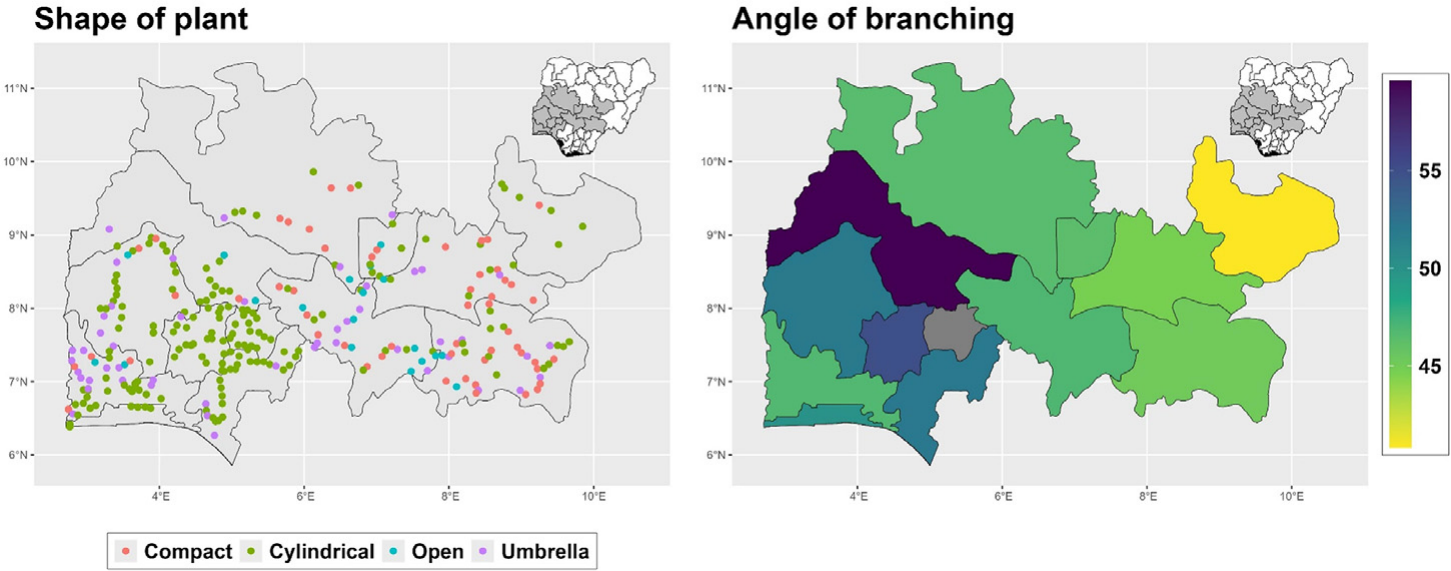
Variations in agromorphological traits at 12 months.

**Fig. 17:**
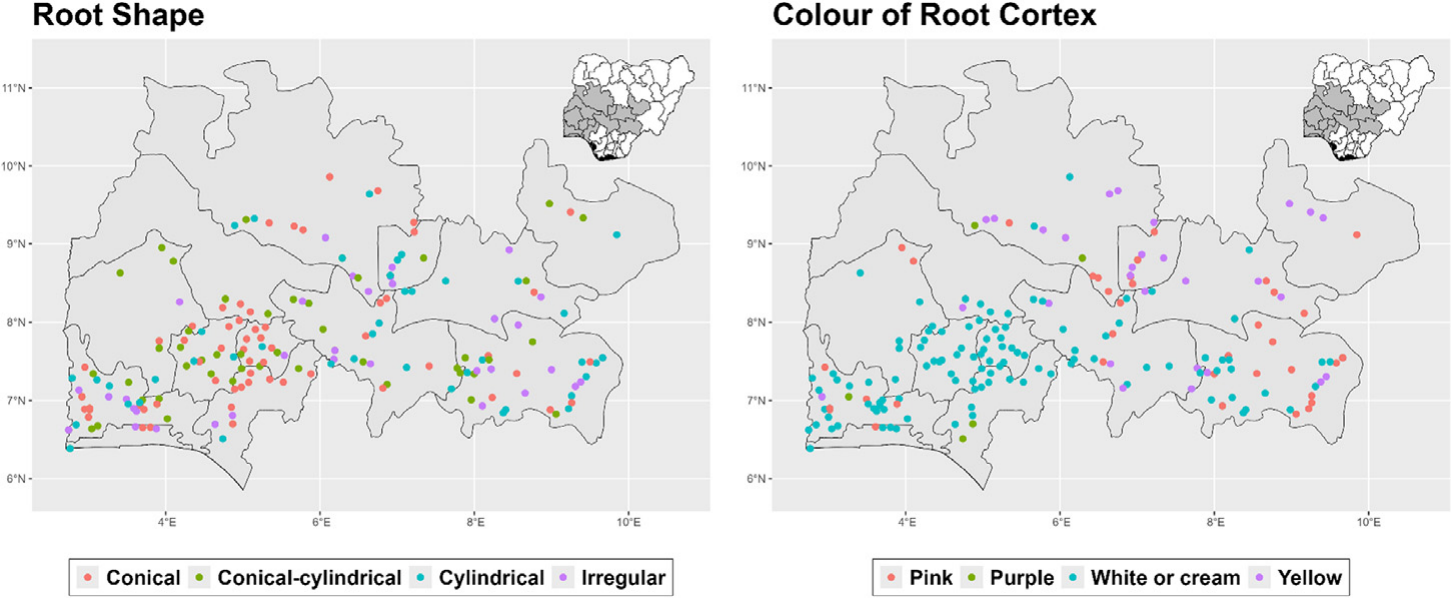
Variations in agromorphological traits measured at Harvest.

**Fig. 18:**
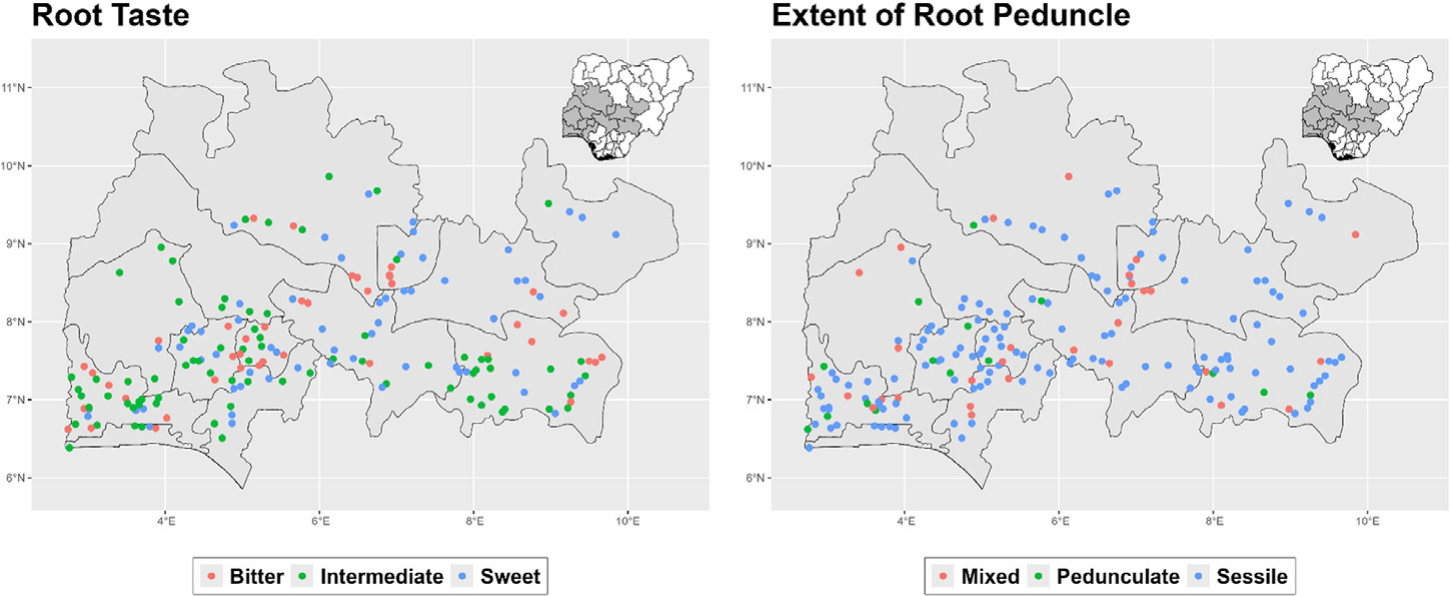
Variations in agromorphological traits measured at Harvest.

**Fig. 19:**
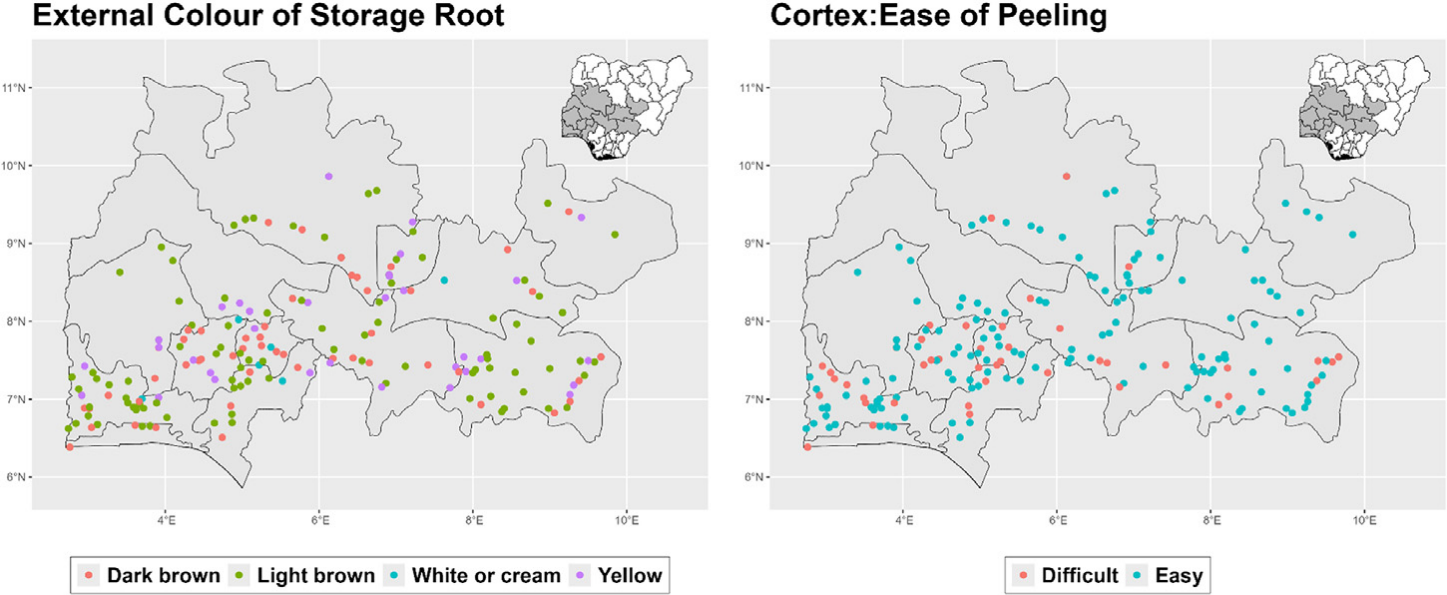
Variations in agromorphological traits measured at Harvest.

**Fig. 20:**
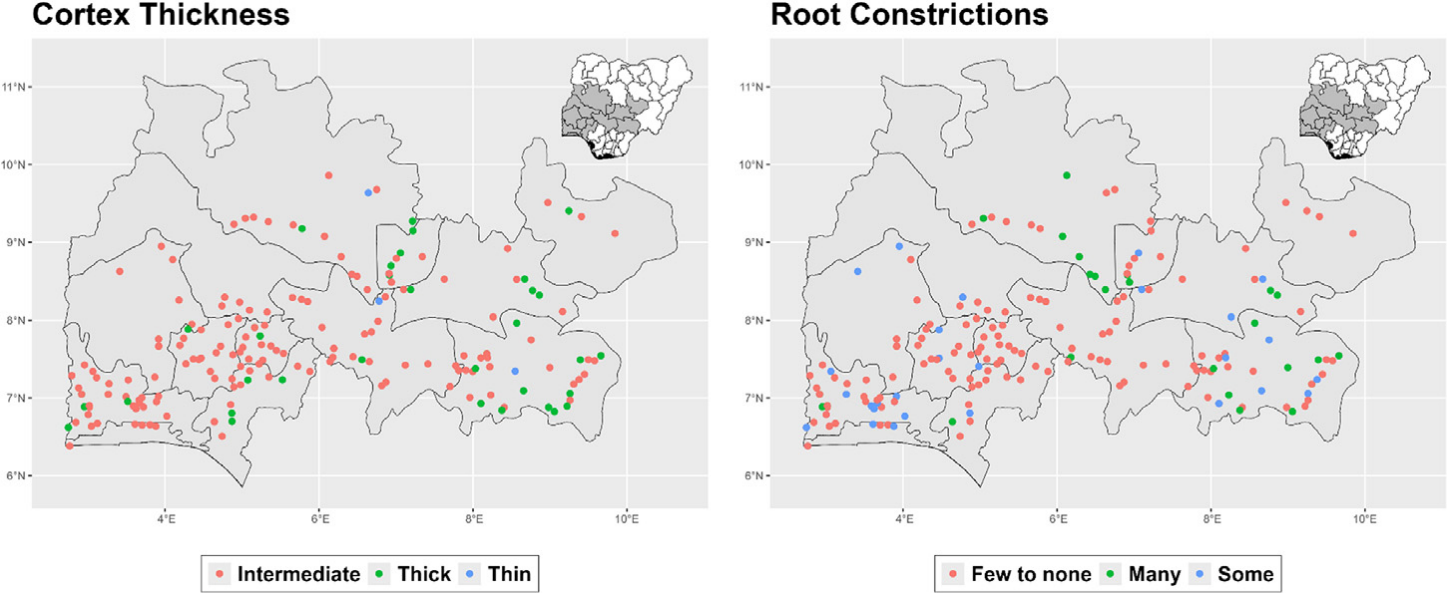
Variations in agromorphological traits measured at Harvest.

**Fig. 21:**
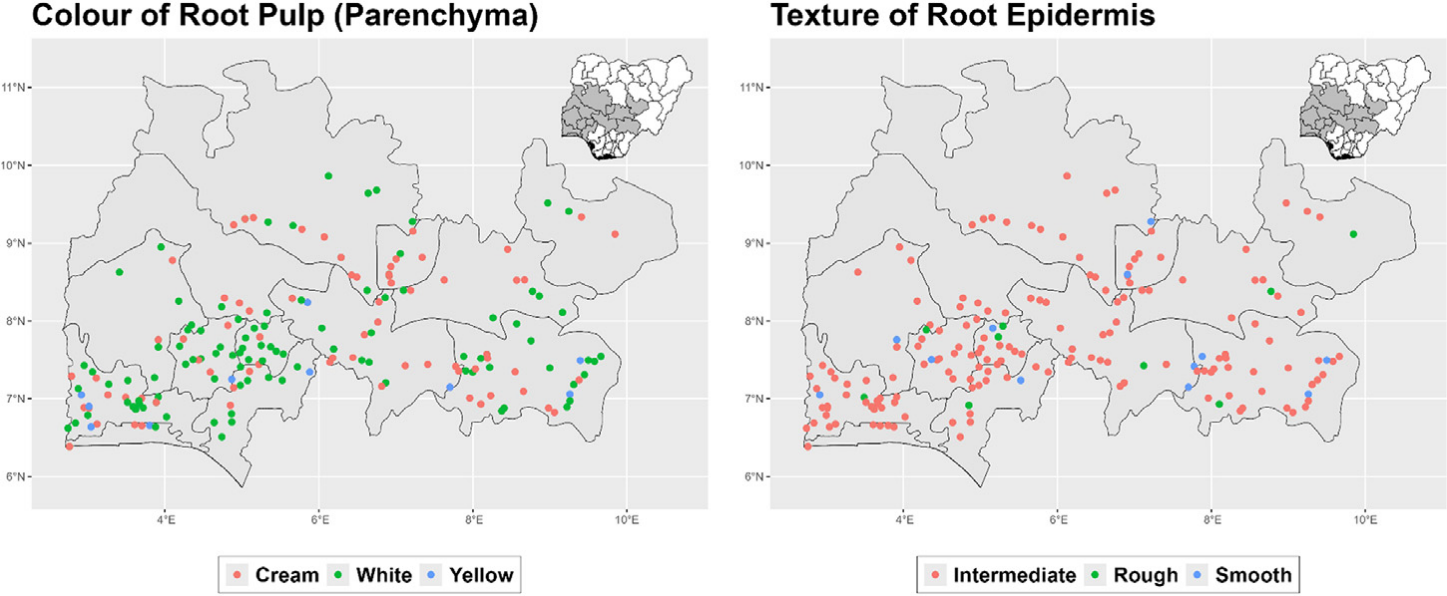
Variations in agromorphological traits measured at Harvest.

**Fig. 22:**
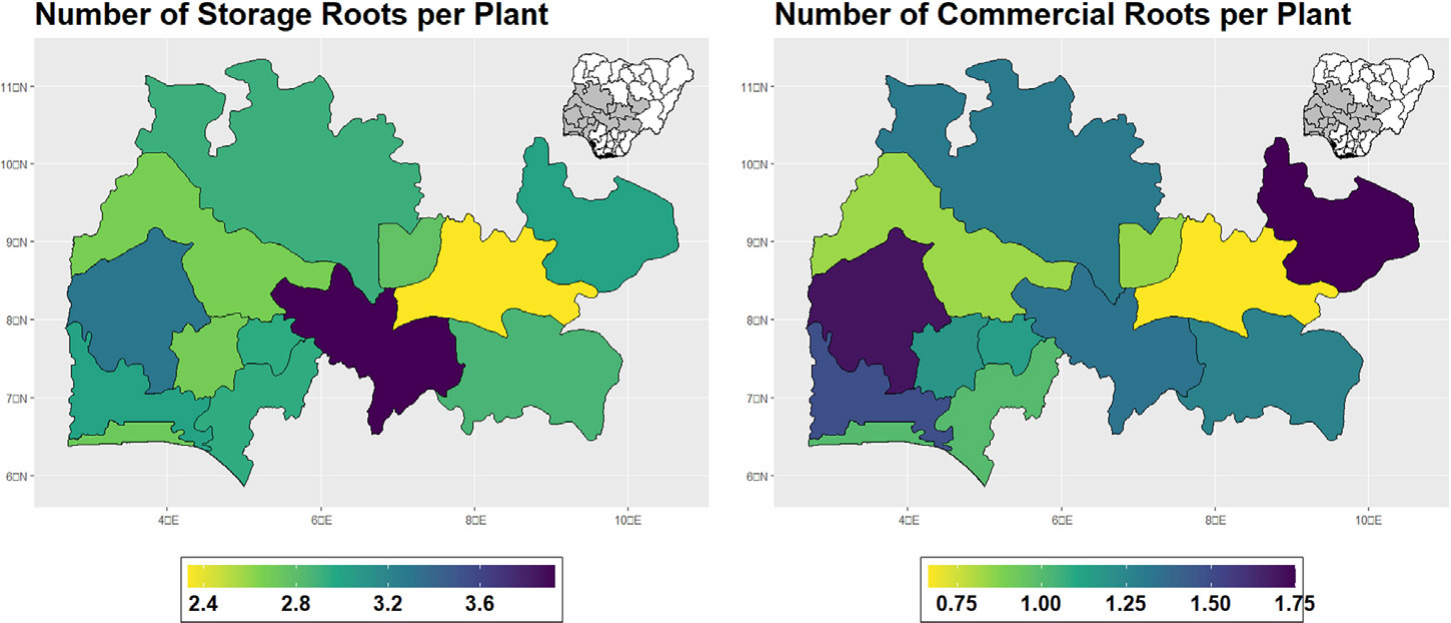
Variations in agromorphological traits measured at Harvest.

**Fig. 23:**
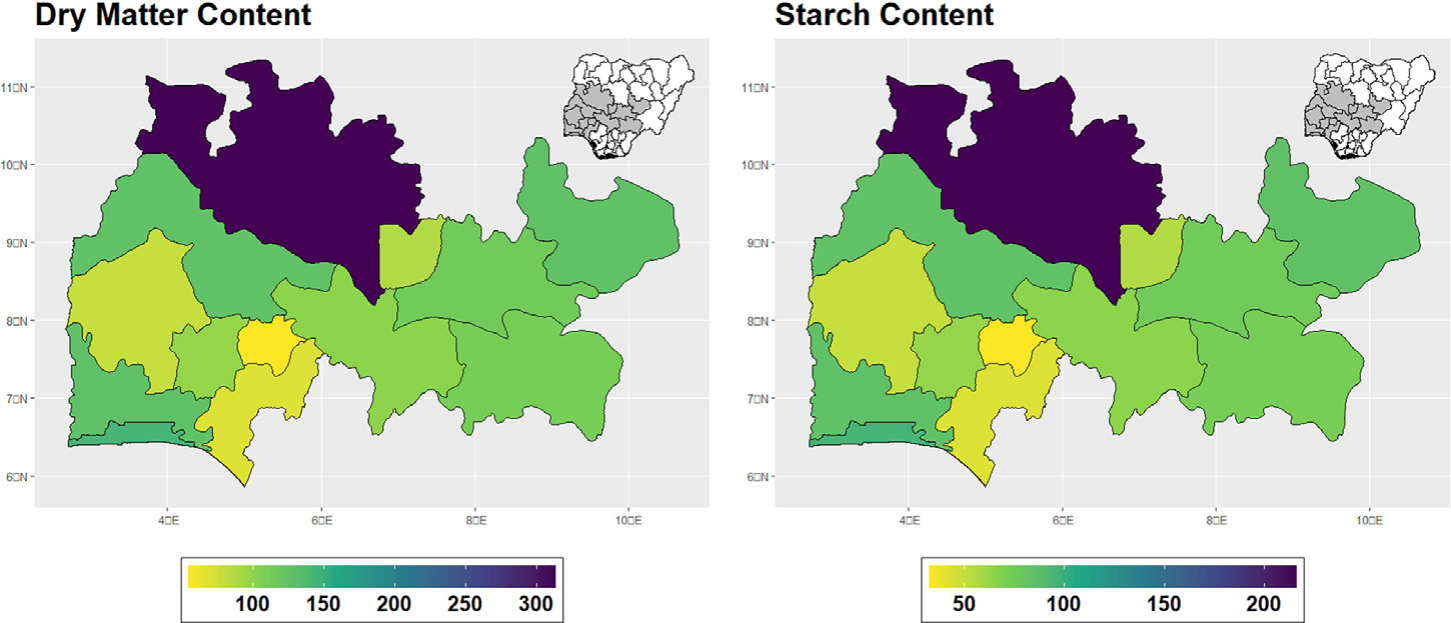
Variations in agromorphological traits measured at Harvest.

**Fig. 24:**
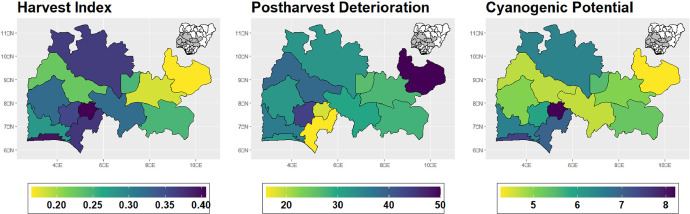
Variations in agromorphological traits measured at Harvest.

**Fig. 25:**
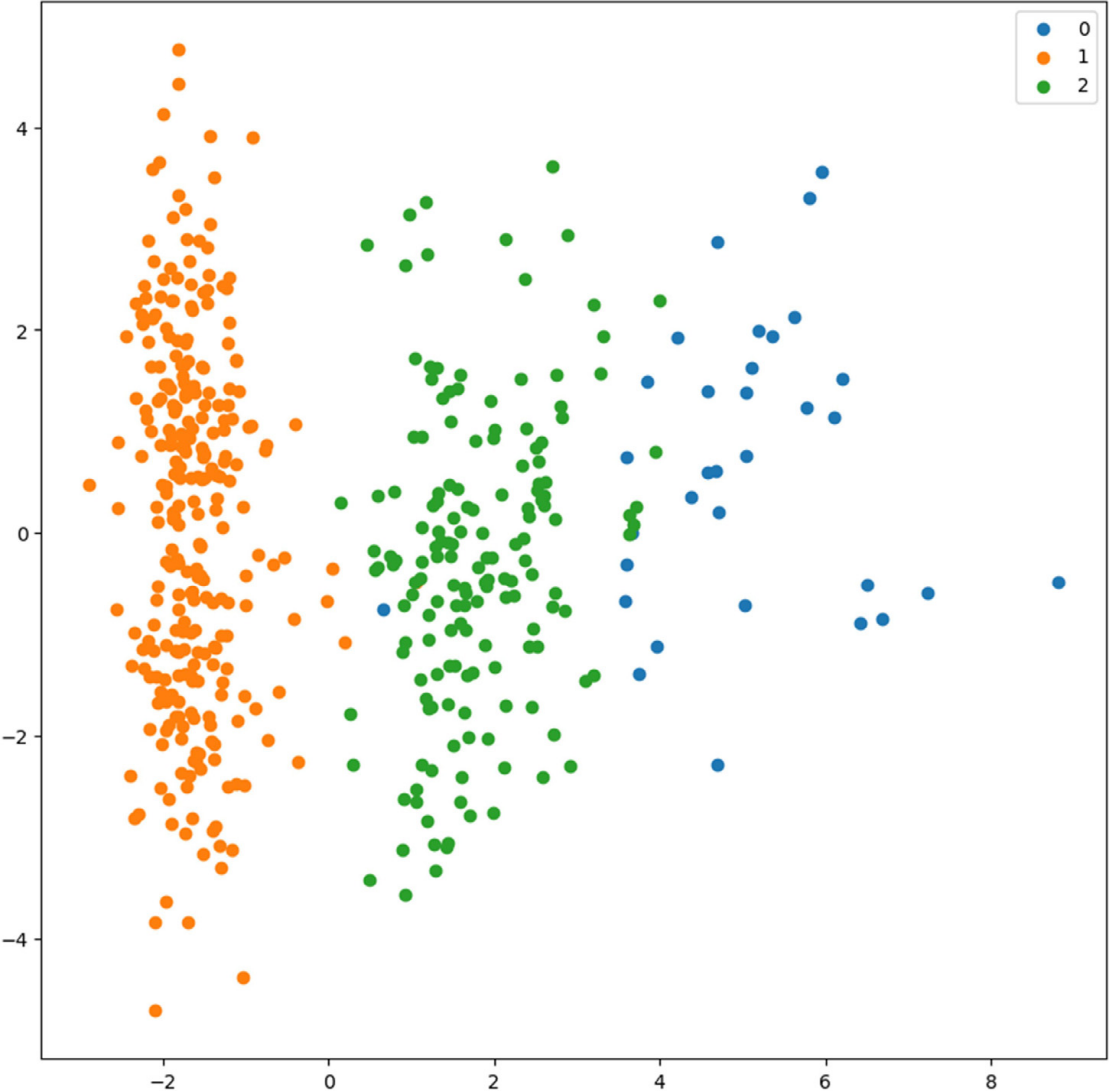
Scatter plot showing that all accessions cluster into 3 main groups based on pre-harvest information.

**Fig. 26:**
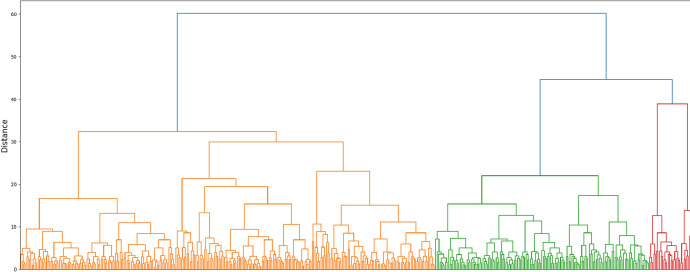
Hierarchical clustering dendrogram of obtained accessions into three groups.

## 4 Experimental Design, Materials and Methods

### 4.1 Sample collection

The data presented here represents agromorphological descriptors of 470 cassava accessions obtained during routine field surveys across Southwest and North Central Nigeria between November 2021 and January 2022 as shown in Fig. 1 above. The survey was conducted along a predetermined route with farms surveyed a minimum of 10km apart as previously described by (Sseruwagi et al., 2004) [2]. On each surveyed farm, unique cassava accessions were determined by accessing the color of apical leaves, petiole color, leaf color, number of leaf lobes, color leaf vein and prominence of foliar scars. A representative sample of each unique cassava accession was collected and stored in a bucket containing water to prevent drying.

### 4.2 Data collection

A total of 470 cassava accessions were planted at the at the research field of the Central and West Africa Virus Epidemiology (WAVE) program Covenant University hub, in a completely randomized design. The field was maintained using good agronomic practices to ensure cleanliness and facilitate adequate growth and development of the cassava plants. Agromorphological descriptors of obtained cassava accessions were collected following the method of (Fukuda et al., 2010) [1]. data were collected intermittently at three months, six months, nine months and at harvest. A list of characteristics measured are presented in the Table 1, Table 2, Table 3 below.

**Table 2:** Qualitative Traits and corresponding variations observed at 3,6,9 MAP.

Traits to be observed	Description	Variation
Traits observed 3 months after planting		
Colour of Apical leaves	Observations were made and the most frequent occurrence was recorded.	(3) Light green; (5) Dark green; (7) Purplish green; (9) Purple
Pubescence on Apical leaves	Observations were made and the most frequent occurrence was recorded.	(0) Absent; (1) Present
**Traits observed 6 months after planting**		
Leaf retention	Visually scored for leaf retention using a scale of 1–5. An average plant is one with leaves covering about half of the plant. Observations were made and the most frequent occurrence was recorded.	(1) Very poor retention; (2) Less than average retention; (3) Average leaf retention; (4) Better than average retention; (5) Outstanding leaf retention
Shape of central leaflet	Leaf taken from a mid-height position. Observations were made and the most frequent occurrence was recorded.	(1) Ovoid; (2) Elliptic-lanceolate; (3) Obovate-lanceolate; (4) Oblong-lanceolate; (5) Lanceolate; (6) Straight or linear; (7) Pandurate; (8) Linear-piramidal; (9) Linear-pandurate; (10) Linear-hostatilobalate
Petiole color	Leaf taken from a mid-height position. Observations were made and the most frequent occurrence was recorded.	(1) Yellowish-green; (2) Green; (3) Reddish-green; (5) Greenish-red; (7) Red (9) Purple
Leaf color	Observed a leaf from the middle of the plant. Observations were made and the most frequent occurrence was recorded.	(3) Light green; (5) Dark green; (7) Purple green; (9) Purple
Lobe margins	Observed from the middle third of the plant. Observations were made and the most frequent occurrence was recorded.	(3) Smooth; (7) Winding
Color of leaf vein	Observed near the base of the lobes, on the upper side of the leaf, on the central lobe from a leaf from the middle of the plant. Observations were made and the most frequent occurrence was recorded.	(3) Green; (5) Reddish-green in less than half of the lobe; (7) Reddish-green in more than half of the lobe; (9) All red
Number of leaf lobes	Observed a leaf from the middle of the plant. Assessed five leaves and took the predominant number of lobes.	(3) Three lobes; (5) Five lobes; (7) Seven lobes; (9) Nine lobes; (11) Eleven lobes
Flowering	At least one flower on each plant was observed, Scoring was done at repeated regular intervals until harvest to determine whether flowering occurred.	0 Absent 1 Present
Orientation of petiole	Observed from the middle of the plant. Observations were made and the most frequent occurrence was recorded.	(1) Inclined upwards; (3) Horizontal; (5) Inclined downwards; (7) Irregular
**Traits Observed at 9 Months after planting**		
Prominence of foliar scars	Observed from the middle third of the plant. Observations were made and the most frequent occurrence was recorded.	(3) Semi-prominent (5) Prominent
Color of stem cortex	Observed from the middle third of the plant. A small shallow cut and peel back of the epidermis was made, Observations were made, and the most frequent occurrence was recorded.	(1) Orange; (2) Light green; (3) Dark green
Color of stem epidermis	The back of the epidermis was peeled and the underside of the of the epidermis was observed.	(1) Cream; (2) Light brown; (3) Dark brown; (4) Orange
Color of stem exterior	Observed on the middle third of the plant. Observations were made and the most frequent occurrence was recorded.	(3) Orange; (4) Greeny-yellowish; (5) Golden; (6) Light brown; (7) Silver (8) Gray; (9) Dark brown
Growth habit of stem	Observations were made and the most frequent occurrence was recorded.	(1) Straight; (2) Zig-zag
Distance between leaf scars	Measured from the middle of stem on the middle third of the plant, where the scars are not flat. A measurement was made along the stem then divided the distance by the number of nodes in the measured part.	(3) Short ≤ (8 cm); (5) Medium (8–15 cm); (7) Long ≥ (15 cm)
Color of end branches of adult plant	Observation was taken on top 20 cm of the plant. Observations were made and the most frequent occurrence was recorded.	(3) Green; (5) Green-purple; (7) Purple
Stipule margin	Observed from the upper third of the plant. Observations were made and the most frequent occurrence was recorded.	(1) Entire; (2) Split or forked
Length of stipules	Observed from upper third of plant. Observations were made and the most frequent occurrence was recorded.	(3) Short; (5) Long

**Table 3:** Qualitative traits and corresponding variations to be observed at harvest (12 MAP).

Traits to be observed	Descriptions	Variations
Branching habit	Observed at the lowest or first branching. Recorded the most frequent occurrence on the plot.	1 Erect 2 Dichotomous 3 Trichotomous 4 Tetrachotomous
levels of branching	Recorded number of branching divisions. Zero (0) for no branching	-
Shape of plant	Observations were made and the most frequent occurrence was recorded.	1 Compact 2 Open 3 Umbrella 4 Cylindrical
Extent of root peduncle	Main roots only. Observations were made and the most frequent occurrence was recorded.	0 Sessile 3 Pedunculate 5 Mixed
Root constrictions	Measured only on mature root. Observations were made and the most frequent occurrence was recorded.	1 Few to none 2 Some 3 Many
Root shape	Observations were made and the most frequent occurrence was recorded.	1 Conical 2 Conical-cylindrical 3 Cylindrical 4 Irregular
External Colour of storage root	Observations were made and the most frequent occurrence was recorded.	1 White or cream 2 Yellow 3 Light brown 4 Dark brown
Colour of root pulp (Parenchyma)	Observations were made and the most frequent occurrence was recorded.	1 White 2 Cream 3 Yellow 4 Orange (no photo) 5 Pink
Colour of root cortex	Observations were made and the most frequent occurrence was recorded.	1 White or cream 2 Yellow 3 Pink 4 Purple
Cortex: ease of peeling		1 Easy 2 Difficult
Texture of root epidermis	Record the most common root type.	3 Smooth 5 Intermediate 7 Rough
Cortex thickness	Measured from three roots, at the proximal (closest to stem), mid- and distal (furthest from stem) ends.	1 Thin 2 Intermediate 3 Thick
Root taste	Observations were made on raw roots only and the most frequent occurrence was recorded.	1. Sweet 2. Intermediate 3. Bitter
Flowering	Observations were made and the most frequent occurrence was recorded.	0. Absent 1. Present
Seed	Observations were made and the most frequent occurrence was recorded.	0 Absent 1 Present
Fruit	Observations were made and the most frequent occurrence was recorded.	0 Absent 1 Present

**Table 4:** Quantitative traits measured at 6 and 12 MAP and their code.

Period	Traits to be observed	Descriptions
	Length of leaf lobe	Measured two leaves from the middle of the plant. Measured from the intersection of all lobes to the end of the middle lobe. Expressed in cm and recorded to one decimal place.
	Width of leaf lobe	Measured two leaves from the middle of the plant. Measured from the widest part of the middle lobe. Expressed in cm, and recorded to one decimal place.
	Ratio: length lobe/width lobe (cm)	This was calculated using the lobe length and width lobe.
	Petiole length	Observed from the middle third of the plant. Measured two leaves/ plant. Expressed in cm.
12 MAP	Height of plant	Measured vertical height from the ground to the top of the canopy. Expressed in cm.
	Angle of Branching	Measured at first primary branching (not side branches). The measured angle was divided by two and recorded.
	Height to first Branching	Measured vertical height from ground to first primary branch. Zero = no branching. Expressed in cm.
	Number of Commercial roots per plants	Recorded the number of roots with length greater than 20 cm.
	Number of storage roots per plant	Observations were made and the most frequent occurrence was recorded.
	Weight of roots per plant	weight of freshly harvested roots
	Weight of fresh aboveground biomass per plant	weight of aboveground biomass of freshly harvested roots
	Harvest index	Measured on each plants

### 4.3 Determination of cyanogenic potential

Cyanide's potential was estimated as described by (Fukuda et al., 2010) [1]. Briefly, a cross-sectional cut was made at the mid-point position of each cassava root and collected into glass tubes. Five 5 drops of toluene were added to each tube after which picrate saturated Whatman filter paper was suspended above the cassava sample within the glass tube. The glass tube was tightly covered and allowed to sit for 12 hours following which the colour intensity of the filter paper was assessed on a scale of 1-9 with a score of 1 indicating low cyanogenic potential, while 9 denoted high cyanogenic potential.

### 4.4 Determination of post-harvest deterioration

Post-harvest deterioration was determined on roots measuring at least 18cm in length. 1cm of the proximal and distal ends of the roots were trimmed, cling film was used to cover the distal end and the roots were stored under ambient condition. After a seven-day period, 2cm transversal slices were made starting from the proximal end of each root and scored on a scale of 1-10 based on the degree of discoloration observed on the cut surface, with 1 denoting minimum deterioration, where about 10% if the slices affected, while a score of 10 indicated complete deterioration of the entire root.

### 4.5 Determination of harvest index

Harvest Index was calculated by weighing the total roots harvested from each accession and weighing the aboveground biomass (stems, branches, and leaves). Mathematically, HI was computed as HI = weight of roots/ (weight of roots + weight of aboveground biomass).

### 4.6 Determination of dry matter and starch content

This was determined by the method described by (Fukuda et al., 2010) [1], dry matter content and starch content have a linear relationship with specific gravity (X). The Percentage of dry matter content was determined using the formula DM = 158.3x – 142, while starch content was calculated as SC = 112.1x – 106.4; where ‘X’ is the specific gravity. The specific gravity was determined following this method: roots were carefully cleaned to remove soil and other debris. Clean root samples from each accession were weighed in air (Wa) using weighing scale, thereafter, weighed in water (Ww). Specific gravity (x) was computed using this formula Ww/Wa – Ww.

## Limitations

None.

## Ethics Statement

The authors have read and follow the ethical requirement for publication in Data in Brief and confirm that the current work does not involve human subjects, animal experiments, or any data collected from social media platforms.

## CRediT authorship contribution statement

**Isaac O. Abegunde:** Data curation, Formal analysis, Writing – original draft. **Oghenevwairhe P. Efekemo:** Investigation. **Olabode Onile-ere:** Investigation, Writing – review & editing. **Folashade Otitolaye:** Investigation. **Emmanuel O. Idehen:** Writing – review & editing. **Angela O. Eni:** Conceptualization, Methodology, Funding acquisition, Writing – review & editing.

## Data Availability

Datasets on agromorphological characters and distribution of cassava accessions cultivated in South-West and North-Central regions of Nigeria. (Original data) (Mendeley Data).
